# Statistical analysis plan for Love Your Brain: a multi-arm randomised controlled trial of a stroke prevention digital platform

**DOI:** 10.1186/s13063-026-09548-z

**Published:** 2026-02-25

**Authors:** M. F. Kilkenny, S. L. Gall, D. A. Cadilhac, A. G. Thrift, M. R. Nelson, J. Bray, J. Cameron, T. Kleinig, L. Murphy, T. Purvis, R. Freak-Poli, C. Burns, C. Farmer, B. Bullas, L. L. Dalli, E. Horton, B. Booth, S. Ho, M. T. Olaiya

**Affiliations:** 1 Stroke and Ageing Research, Department of Medicine, School of Clinical Sciences at Monash Health, Monash University, Clayton, VIC Australia; 2 Stroke and Critical Care Theme, Florey Institute of Neuroscience and Mental Health, University of Melbourne, Heidelberg, VIC Australia; 3 Menzies Institute for Medical Research, University of Tasmania, Hobart, TAS Australia; 4 School of Public Health and Preventive Medicine, Monash University, Melbourne, VIC Australia; 5 Department of Neurology, Royal Adelaide Hospital, Adelaide, SA Australia; 6 Department of Medicine, University of Adelaide, Adelaide, SA Australia; 7 Stroke Foundation, Melbourne, VIC Australia

**Keywords:** Stroke, Digital health, EHealth, Statistical analysis plan, Randomized controlled trial

## Abstract

**Background:**

Stroke is common, affecting an estimated one in four people in their lifetime. Fortunately, stroke is also highly preventable. With the rise in digital literacy and the increasing adoption of digital health tools, a promising avenue for stroke prevention is through digitally-delivered interventions. *Love Your Brain: A stroke prevention digital platform* is a three-year research project to develop and evaluate a digital platform to 1) increase participant visits to their medical practitioner for assessment or management of cardiovascular risk factors (primary outcome); and 2) improve participants’ health-related stroke knowledge, adherence to prevention medications and uptake of healthy or risk-modifying behaviour (secondary outcomes). The project is a multi-arm randomised controlled trial, comparing the common control arm to each of the intervention arms (either an online course or text messages). We present the statistical analysis plan for this trial.

**Methods/design:**

Participants are randomised 1:1:1 in variable block sizes, with stratification balancing by age and sex. The sample size of 894 participants was calculated to detect a 30% relative intervention effect, with 80% power and 5% significance level (two-sided). Recruitment will end when the sample size is achieved (adjusting for ≤ 10% attrition rate). The primary outcome will be compared separately for each intervention arm with the common control arm. As the outcome is binary, log-binomial regression models will be used with adjustment for stratification variables (i.e., gender, age groups [45–64 and >= 65 years]) and covariates that demonstrate imbalances between arms at baseline. Secondary outcomes will be evaluated using generalised mixed effects regression models (including linear, log-binomial, or quantile regression). The primary outcome analysis will be based on intention-to-treat. A p-value ≤ 0.05 will indicate statistical significance.

**Conclusions:**

This statistical analysis plan ensures transparency in reporting the trial outcomes. Love Your Brain will provide novel evidence on the effectiveness of two digital health education platforms for the prevention of stroke.

**Trial registration:**

ACTRN12625000124437; U1111-1305-2964

Feasibility Pilot: Australian New Zealand Clinical Trials Registry: ACTRN12624000540516; Universal Trial Number: U1111-1305-2964.

SAP Version: 1.0 (September 2025)

Protocol version: 1.0 (December, 2024)

SAP revisions: Nil

**Supplementary Information:**

The online version contains supplementary material available at 10.1186/s13063-026-09548-z.

## Introduction

Stroke is common, affecting an estimated one in four people in their lifetime [1]. In 2023, the direct costs of stroke to the Australian economy was $9 billion, with an expected lifetime cost exceeding $15 billion per person with stroke [2]. Fortunately, stroke is also highly preventable. Of the 45,000 strokes that occur in Australia each year [2], ≥ 36,000 are estimated to be preventable through effective management of risk factors, such as smoking, poor quality diet, high blood pressure and physical inactivity [3].

Stroke Foundation aims to raise awareness of stroke in Australia. Their StrokeSafe program involves recruiting volunteers to present to local communities about signs, symptoms, and risk factors of stroke. The goals are two-fold: 1) to enhance knowledge about how to prevent stroke, the early signs/symptoms of stroke; and 2) to seek immediate medical attention when signs of stroke are observed, which can significantly improve outcomes [4]. The StrokeSafe program has delivered over 6,500 presentations since 2010, reaching over 190,000 people. The StrokeSafe presentations are mostly delivered by people with lived experience of stroke. They are live (online or in-person) and delivered in a group setting. The StrokeSafe presentations have been effective in improving knowledge of stroke risk factors and signs of stroke [5]. However, Kilkenny et al. [5] showed that this knowledge diminished after three months, identifying the importance of continued exposure to information to improve knowledge retention.. Increasing community knowledge of the major risk factors for stroke, such as high blood pressure and diabetes, can result in behavioural change [5].

With the rise in technology literacy and the increasing adoption of digital health tools, a promising avenue for stroke prevention is through digitally delivered interventions [6, 7]. This approach offers several potential benefits, including increased accessibility, personalised information, and the ability to reach a wide audience. *Love Your Brain: A stroke prevention digital platform* is a 3-year research project to develop and evaluate, using a randomised controlled trial (RCT) design, a digital platform that includes different health promotion education strategies that aim to prevent stroke through behaviour change and improve health knowledge. It is a collaboration between Monash University, Menzies Institute for Medical Research (University of Tasmania), and Stroke Foundation.

The project comprises a multi-arm randomised controlled trial (RCT), comparing the common control arm to each of the intervention arms (either an online course or text messages). The Love Your Brain digital platform aims to motivate behaviour change for stroke prevention and improve stroke knowledge. The primary aim is to determine the efficacy of the digital platform in improving attendance at a medical practitioner (including a general practitioner or specialist), for cardiovascular risk assessment or management, compared to a common control arm at 12 weeks post-randomisation. We present the statistical analysis plan for the Love Your Brain trial.

## Methods

**Fig. 1:**
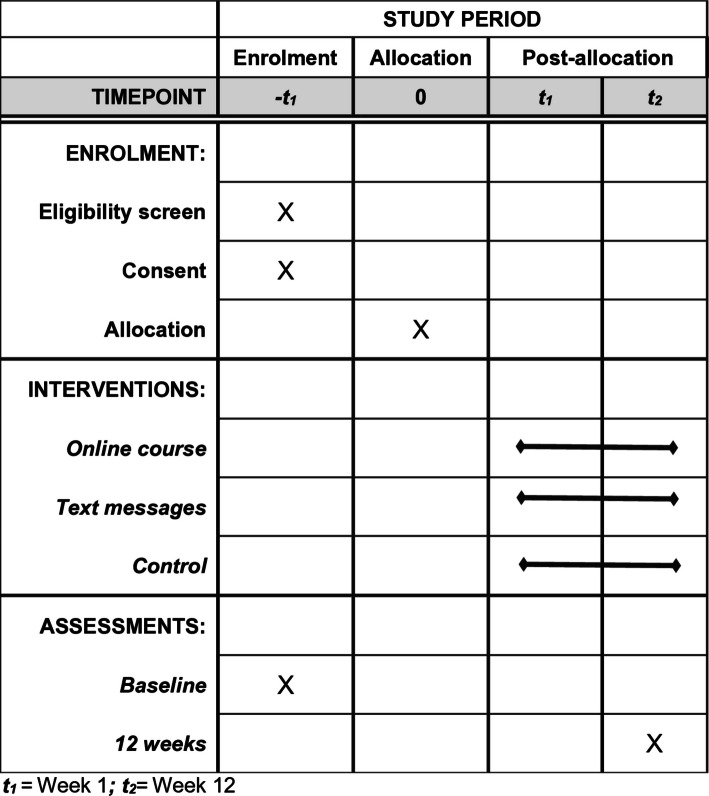
SPIRIT figure

This statistical analysis plan has been written according to the “Guidelines for the Content of Statistical Analysis Plans in Clinical Trials” [8]. The trial protocol has been described in detail previously [9] and is briefly outlined in the sections below. In addition, details of the initial trial design (Fig. 1), and any subsequent changes made, have been published on the Australian and New Zealand Clinical Trials Registry (ACTRN12625000124437). This statistical analysis plan comprises details of our pre-planned statistics to be used for the analysis of primary and secondary outcomes.

### Trial design

This is a prospective, multi-arm RCT, with a 1:1:1 allocation ratio, and intention-to-treat analysis.

### Randomisation and blinding

Randomisation is undertaken through the REDCap online system [10–12], with stratification balancing by age (45–64, ≥ 65 years) and gender (man, woman, non-binary/gender diverse/prefer not to say). The randomisation table, comprising the allocation sequence, block sizes, and stratification balancing, was developed outside of REDCap by an independent data analyst and imported into the REDCap database.

The trial has a single blinded study design. Participants are unblinded to arm allocation. The trial is described in the participant information and consent form in general terms as involving a “digital platform”, with information shared via email, text messages, and online course. The trial biostatistician undertaking the analysis and research staff who interact with participants at the 12-week follow-up, are blinded to arm allocation.

### Sample size calculation

The sample size was based on a conservative estimate of 30–40% prevalence of general practitioner health checks (the primary outcome) in Australia [13, 14]. We estimated a sample size of 894 participants (~ 298 participants per arm) to allow for ≥ 80% power, and α 0.05 to detect a 30% relative increase in the proportion of participants undertaking health checks, and ≤ 10% attrition rate based on trials of similar intervention intensity [15]. Using a model from the UK, increases of this magnitude in attending health checks are estimated to have meaningful effects on population-level health and economic outcomes [16]. Based on Howard et al., [17] we are using a common control arm with no adjustment for family-wise type-I error rate because our hypotheses do not inform a common claim of effectiveness for each of the interventions.

We will cease recruitment when sufficient participants are recruited (including adjusting for the actual attrition rate after randomising 600 participants). The number of participants obtained will be used without any re-estimation of power.

### Framework

All outcome analyses will be conducted to determine the effectiveness of individual Love Your Brain intervention arm over the control arm (described in detail below).

### Statistical interim analyses and stopping guidance

Not applicable.

### Reporting of adverse events

The Love Your Brain Project Coordinator is notified by email each time an adverse event is self-reported by participants. A blinded member of the research team will assess the severity, and relationship to study treatment in REDCap. Any serious adverse events deemed likely to be related to the trial intervention will be independently adjudicated by a medical monitor independent of the trial. Serious adverse events not related to the intervention will be reported annually to the ethics committee in a summary table. The detailed definitions of adverse events are published in our prior protocol [9].

### Timing of outcome assessments and final analysis

Participants are invited to complete the primary outcome assessment (self-reported medical practitioner visit) and secondary outcome assessments (stroke knowledge, health behaviour change and medication adherence) at 12 weeks after randomisation (with 4 weeks allocated to complete these; Table 1 and Fig. 1) [9]. The 12-week duration was selected based on evidence from habit-formation research and behaviour-change interventions [18]. Outcome analyses will commence after all participant assessments and evaluations are completed.

**Table 1:** List of outcome variables collected in the trial

Self-reported outcome assessment	Baseline	12-week
**Primary outcome**		
Medical practitioner visit for cardiovascular risk assessment or management, from general practitioner or specialist	✓	✓
**Secondary outcomes**		
Stroke knowledge	✓	✓
Health behaviour change		
Physical activity	✓	✓
Diet	✓	✓
Body-mass index	✓	✓
Smoking cessation	✓	✓
Alcohol consumption	✓	✓
Sleep and wellbeing	✓	✓
Medication adherence^a^	✓	✓
**Process and economic evaluation**		
Satisfaction and evaluation survey		✓
Spend on health behaviours^b^	✓	✓
Healthcare resource utilisation^c^	✓	✓
Interviews with participants		✓

^a^For participants who indicate current prescription medication use

^b^For example, expenses on memberships or class fees for physical activity

^c^For example, visiting a general practitioner or allied health practitioner, validated through linkage with administrative data

## Statistical principles

### Confidence intervals and p-values

Statistically significant results will be those estimated using two-sided tests, at a 5% significance level. Estimates of this study will be reported with 95% confidence intervals.

### Adherence and protocol deviations

Intervention fidelity are being assessed throughout the trial. This includes monitoring recruitment procedures, the dispatch logs from the electronic messaging gateway, the online course gateway, and the completion rate of the 12-week completion survey. The intervention fidelity procedures have been developed to address four key areas of the study: (1) study design; (2) training documents and processes; (3) delivery of the Love Your Brain interventions (e.g. failure to receive any messages or not commencing the online course); and (4) receipt of intervention (e.g. audit of dispatch logs; participation Supplemental Table I). This approach is consistent with the Behaviour Change Consortium treatment fidelity recommendations [19]. Deviations related to fidelity are considered minor (non-serious). Other examples include (a) missed or incomplete study procedure (e.g. component of a survey is skipped, email fails to send); (b) study procedure completed outside protocol timeframe (e.g., completion of 12-week survey after final access date); and (c) unblinding of research assistant interacting with participants.

Any participant treated in a manner that deviates from the protocol may be excluded from per-protocol analyses. The nature and reasons for any protocol deviation are recorded in the REDCap.

All deviations related to intervention delivery (e.g., timing, missed messages) will be documented and summarised descriptively by type and severity. Fidelity metrics will not affect the primary intention-to-treat analysis. However, they will inform the definition of the per-protocol population detailed in additional analyses. A separate table will provide details of fidelity metrics, including the number, proportion, and nature of protocol deviations.

### Analysis populations

Analysis of the primary outcome will be based on the principle of intention-to-treat and will comprise all randomised participants.

Further per-protocol analyses will be undertaken among participants who complete at least 50% of the intervention arm to which they were randomised [14]. This involves completing all two core modules and at least two of the additional modules for participants receiving the online course, and receiving text messages for 6 of 12 weeks for the text messages arm [20].

## Trial population

### Screening, eligibility, and recruitment

**Fig. 2:**
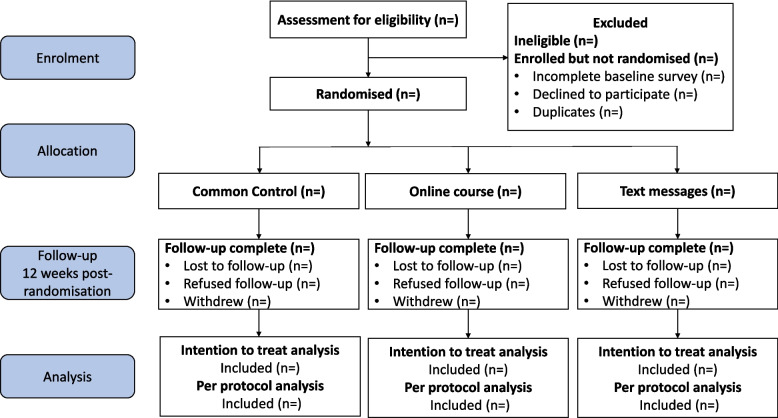
CONSORT flow diagram

To determine the representativeness of the trial cohort and external validity of the trial findings, we will compare characteristics of Love Your Brain participants (e.g. age, gender and location) with the general population using data from the Australian Bureau of Statistics Census Survey [21]. The details of recruitment and eligibility criteria are described in the trial protocol [9], and information on eligibility, recruitment, and withdrawal/follow-up will be reported in a CONSORT flow diagram (Fig. 2). Outcome assessments are integrated into the trial using existing systems with automated email distribution. Using the Dillman protocol [22], three attempts at contact (two emails and at least one telephone follow-up) will be made to improve response rates for outcome assessments, as described in detail in the protocol [9].

### Baseline participant characteristics

Participant characteristics will be collected at baseline and includes demographic, socio-economic factors (e.g. educational attainment), cardiovascular risk factors and digital platform content preferences. Data on baseline characteristics (i.e. demographics, cultural or ethnic group, health literacy, and self-reported medical history) will be summarised as frequencies and proportions for categorical variables, and medians and interquartile ranges for continuous variables (Table 2).

**Table 2:** Baseline characteristics of the online course or text message intervention arms *vs*. control arm

Baseline characteristics	Control *N* = *n* (%)	Online course *N* = *n* (%)	Text messages *N* = *n* (%)	Control vs online *P*-value	Control vs text messages *P*-value
**Demographics**					
Median age (interquartile range), in years					
45–64					
65 +					
Female					
University educated					
Employed					
**Cultural or ethnic group**					
Australian					
Australian Aboriginal					
New Zealander					
British					
Irish					
Western European					
Northern European					
Southern European					
South-East Asian					
Central Asian					
South American					
North American					
Other					
**Health literacy**^a^					
Needs help with health information					
**Self-reported medical history**					
High blood pressure					
High cholesterol					
Atrial fibrillation					
Diabetes/high blood sugar					

Note that not all response options for cultural or ethnic groups are listed

^a^Defined as never or rarely needing help when reading instructions, pamphlets, or other written material from your doctor or pharmacy (Morris NS et. al. BMC Family Practice 2006)

## Analysis

### Outcome definitions

#### Primary outcome

The primary outcome is attendance at a medical practitioner for cardiovascular risk assessment or management, from either a general practitioner or specialist, within 12 weeks of randomisation. Assessment will be via self-report. The primary outcome is based on participants answering affirmatively to the question at 12-week follow-up “Have you ever visited your doctor (general practitioner or specialist) for an assessment or management of your heart and stroke risk factors? This might include your blood pressure, blood glucose, cholesterol, weight, smoking status, or your family’s history of stroke or heart attack” (single response options: Yes; No) and indicating this visit to the doctor was within the last 12 weeks in the subsequent question (“When was this visit to your doctor to check or manage your risk factors?” Single response options: Within the last 12 weeks or less; More than 12 weeks ago). The primary outcome results will be summarised by intervention arm in Table 3. This primary outcome was chosen because it demonstrates behaviour that will lead to risk factor identification and management by a medical practitioner, and this aligns with current prevention objectives of Stroke Foundation. All trial data are being collected via REDCap, with validation checks established to limit implausible or inconsistent response on the primary outcome. The primary outcome analyses are confirmatory.

**Table 3:** Within- and between-group differences in outcomes for the online course or text message intervention arms vs. control arm

Assessment	Control (*N* =)			Online course (*N* =)			Text messages (*N* =)			Online vs control	Text vs control
Self-reported outcomes	BL *n/N* (%)	W12 *n/N* (%)	*P* value	BL *n/N* (%)	W12 *n/N* (%)	*P* value	BL *n/N* (%)	W12 *n/N* (%)	*P* value	RR (95% CI)*	RR (95% CI)*
**Primary outcome**											
Visit to a medical practitioner within 12 weeks for assessment or management of cardiovascular risk factors**											
**Secondary outcomes**											
*Mean stroke knowledge score (SD)*^a^											
*Health behaviour change*											
Physically active^b^											
Healthy diet^c^											
Not overweight^d^											
No current smoking											
Alcohol consumption within guidelines^e^											
Healthy sleep and wellbeing^f^											
Vitality											
Social relationships											
Sleeping well											
Connectedness											
Adherent to medication^g^											

*BL* Baseline, *W12 *12 weeks, *RR *Risk Ratios, *CI *confidence interval, *SD *standard deviation

*Adjusted for gender, age and baseline value;

**Based on participants answering affirmatively to the question “Have you ever visited your doctor (general practitioner or specialist) for an assessment or management of your heart and stroke risk factors? This might include your blood pressure, blood glucose, cholesterol, weight, smoking status, or your family’s history of stroke or heart attack” (single response options: Yes; No) and indicating this visit to the doctor was within the last 12 weeks in the subsequent question (“When was this visit to your doctor to check or manage your risk factors?” Single response options: Within the last 12 weeks or less; More than 12 weeks ago)

^a^Mean percentage of correct responses to the 20-item Stroke Knowledge Test

^b^Physically active is defined as self-reported 30 min or more of moderate-intensity physical activity, or 20 min or more of vigorous-intensity physical activity, at least 3 times a week

^c^Diet score will be determined by the Mini-EATS scoring key with a score above 69 considered healthy

^d^Not overweight is defined as self-reported body mass index of < 25kg/m^2^

^e^Guideline recommended alcohol consumption is defined as < 10 standard drinks per week, and < 4 standard drinks on any day

^f^Healthy sleep and wellbeing is defined as self-reported absence of problems with energy, close relationships, sleep and social isolation

^g^For participants who indicate current use of prescription medication(s), adherence to medication is defined as a score of ≥ 20/25 on the aggregated Medication Adherence Report Scale [21]

#### Secondary outcomes

Secondary outcomes (Table 3) will be changes, from baseline to 12 weeks post-randomisation, in the:

1. Mean percentage of correct responses to the 20-item Stroke Knowledge Test [23, 24]. The stroke knowledge test incorporates assessment of items focused on knowledge of stroke risk, warning signs, and appropriate behaviour.
2. Proportion of participants adhering to healthy or risk-modifying behaviour, measured through valid and reliable self-report questionnaires (e.g. cessation of smoking, increase in physical activity).
3. Proportion of participants adhering to prevention medications, measured using the Medication Adherence Report Scale-5 [25].

More details on these outcomes have been provided previously [9]. The secondary outcome analyses are supportive. The subgroup analyses are exploratory analyses and are hypothesis-generating and will be interpreted cautiously.

#### Economic evaluation

Information on health-related quality of life [26], resource use and out of pocket costs will be obtained from participants for use in an economic evaluation. Information on resource use and out of pocket costs will also be obtained from linked Medicare Benefits Schedule and Pharmaceutical Benefits Scheme data.

#### Process evaluation

Qualitative (participant interviews) and quantitative feedback (satisfaction survey) are being collected to assess the acceptability, feasibility, and potential mechanism of actions. Barriers, enablers, and suggestions for improving the digital platform will also be explored.

Participants were aware of their group allocation, and while it is possible that two participants might discuss intervention content informally (e.g. if they encouraged others they know to do the trial), contamination beyond immediate family and friends is highly unlikely. The possibility of contamination will also be formally assessed as part of the process evaluation.

### Analysis methods

Our analytical approach will be guided by CONSORT reporting guidelines [27] for a stratified RCT design, with a binomial primary outcome and intention-to-treat analysis. Based on these guidelines, we will report effect sizes for all primary and secondary outcomes, accompanied by 95% confidence intervals to indicate precision. For binary outcomes, we will present both relative effect measures (risk ratios) and absolute effect measures, including marginal probabilities (predicted probabilities from the fitted model) and risk differences. This approach ensures transparency and aligns with CONSORT 2025 guidance to provide both relative and absolute estimates of treatment effect. Following the intention-to-treat principle, participants will be analysed according to the arm in which they were allocated, regardless of whether or not they received the intervention or deviated from the protocol. The proposed format for presenting study outcomes is shown in Table 3.

#### Primary outcome

The primary outcome will be compared separately for each intervention arm with the control (Table 3). As the outcome is binary, log-binomial regression models will be used with adjustment for stratification variables (i.e. gender, age groups [45–64 and > 65 years]) and covariates that demonstrate imbalances between arms at baseline. If the log-binomial model fails to converge, we will use a Poisson regression with a log link and robust variance estimation as an alternative, which yields consistent RR estimates. Model diagnostics will include assessment of convergence and verification that predicted probabilities remain within the valid range (0–1). In addition, to the relative effect measures [risk ratios and 95% confidence intervals (CIs)], we will also provide the absolute effect measures including marginal probabilities (predicted probabilities from the fitted model) and risk differences with 95% CIs.

#### Secondary outcomes

Within-group change in secondary outcomes will be determined using McNemar’s test for categorical outcomes, and Wilcoxon sign rank test for count outcomes. Generalised mixed effects regression models, including linear, log-binomial, or quantile, functions depending on the nature and distribution of these outcomes, will be used to compare secondary outcomes between online course or text message arms and the common control arm.

#### Multiplicity adjustment

No adjustment for the family-wise type I error rate will be applied because, although a common control arm is used, each intervention is evaluated against the common control independently. Our hypotheses do not inform a single overarching claim of effectiveness across interventions; therefore, multiplicity adjustment is not required. This approach aligns with recommendations for trials [17] where a single trial includes two distinct intervention arms rather than a unified confirmatory claim.

####  Subgroup and additional analyses

*Validation of primary outcome via data linkage*: Analyses of the primary outcome, a healthcare visit for cardiovascular risk factor assessment or management, will be undertaken using linked Medicare claims data. Trial data will be linked at the person level with these Medicare Benefits Schedule and Pharmaceutical Benefits Scheme datasets (Supplemental Table II) for consenting trial participants for the relevant use of these health assessments before and after randomisation. This will provide indication for potential recall bias for the primary outcome.

Sensitivity analyses of primary outcome: For participants who did not visit a medical practitioner for cardiovascular risk factor assessment or management within the 12 weeks immediately before randomisation, we will undertake between-group comparisons of the primary outcome (Table 4). We will also undertake exploratory analyses to examine change from baseline to 12 weeks in cardiovascular risk assessment and management from a medical practitioner (Supplemental Table III). Although we do not anticipate any imbalance of covariates between the trial arms, we will undertake a sensitivity analysis of the primary outcome model, adjusted for any covariates that are not balanced (based on *p* value ≤ 0.05).

**Table 4:** Cardiovascular risk factor assessment or management during the trial, between the online course or text message intervention arms vs. control arm, among participants who did not visit a medical practitioner in the 12 weeks immediately before randomisation

Primary outcome*	Control *N *=	Online course *N* =	Text messages *N* =	Online vs control	Text message vs control
	*n/N* (%)	*n/N* (%)	*n/N* (%)	RR (95% CI)*	RR (95% CI)*
**Primary outcome**					
Visit to a medical practitioner within 12 weeks for assessment or management of cardiovascular risk factors**					
**Risk assessment undertaken at the visit**					
Measure your blood pressure					
Ask about your family history of stroke or heart attack					
Send you for a blood test for cholesterol, kidney function, and/or blood sugar levels					
2 out of 3 above assessments undertaken					
**Risk management undertaken at the visit**					
Provide you with a care plan for managing your risk factors					

*RR *Risk Ratios, *CI *Confidence interval

*Adjusted for age, gender

**Based on participants answering affirmatively to the question “Have you ever visited your doctor (general practitioner or specialist) for an assessment or management of your heart and stroke risk factors? This might include your blood pressure, blood glucose, cholesterol, weight, smoking status, or your family’s history of stroke or heart attack” (single response options: Yes; No) and indicating this visit to the doctor was within the last 12 weeks in the subsequent question “When was this visit to your doctor to check or manage your risk factors?” Single response options: Within the last 12 weeks or less; More than 12 weeks ago)”

*Per-protocol analyses:* We will undertake per-protocol analyses for all outcomes, i.e. analyses restricted to participants who complete at least 50% of the intervention arm to which they were randomised (described above). This will include participants in the online course arm who completed two core modules and ≥ 2 of 9 additional mini-modules, and participants in the text message arm who received messages for ≥ 6 of the 12 weeks.

*Exploratory analyses based on participant characteristics: *Exploratory analyses will be undertaken to assess the influence of baseline participant characteristics (e.g. age group, gender, health literacy, level of education, level of stroke knowledge) on the primary and secondary outcomes. As the trial may not be powered to detect any interactions of these characteristics with the interventions, the results will be interpreted cautiously. To account for any bias in self-report of health behaviours, in sensitivity analyses, we will adjust outcome regression models for covariates that may be associated with differential reporting, e.g. socio-demographic factors, history of medical conditions associated with these health behaviours, and health literacy.

### Missing data

The primary and secondary outcome analysis will be reported without imputation of missing data (complete case analysis). Where there is > 10% missing data for covariates [28], multivariate imputation by chained equation algorithms will be undertaken. Imputed values will be conditional on observed values of the treatment variable and baseline covariates of the primary outcome, including demographic variables, health literacy, and self-reported medical history [29]. This algorithm will be repeated for up to 20 cycles to ensure convergence of the chained equations [30]. Convergence will be assessed using trace plots of the mean and standard deviation of imputed values across iterations and imputations, and will be considered adequate when trace plots show stability with no systematic trends after burn-in [31].

### Future analyses

Process and economic evaluations, including analyses of linked data, will be reported separately.

### Harms

#### Adverse events

Serious adverse events are defined as any hospitalisations or emergency department visits which occur during the trial. Participants self-report serious adverse events at the 12-week completion survey or at the time of withdrawal (Supplemental Table IV).

### Statistical software

All analyses will be undertaken using StataNow 19.5 MP (StataCorp 2026).

## Supplementary Information

Supplementary Material 1.

## Data Availability

The trial data analysed will be available from the corresponding author on reasonable request.

## Abbreviations

MICE: Multivariate imputation by chained equations
RCT: Randomised controlled trial
REDCap: Research Electronic Data Capture
