# Supplementary material for: Statistical analysis plan for Love Your Brain: a multi-arm randomised controlled trial of a stroke prevention digital platform
Source: Trials. 2026 Feb 25;27:259. doi: 10.1186/s13063-026-09548-z (PMC13041423; doi:10.1186/s13063-026-09548-z)
Supplement: Supplementary file 1 — Supplementary Material 1. [file 13063_2026_9548_MOESM1_ESM.docx]

**Statistical analysis plan for Love Your Brain: A multi-arm randomised controlled trial of a stroke prevention digital platform**

Kilkenny MF, Gall SL, Cadilhac DA, Thrift AG, Nelson MR, Bray J, Cameron J, Kleinig T, Murphy L, Purvis T, Freak-Poli R, Burns C, Farmer C, Bullas B, Dalli LL, Horton E,
Booth B, Ho S, Olaiya MT

**Supplemental Material**

**Supplemental Methods**

**Supplemental Tables**

Supplemental Table I. Intervention fidelity

Supplemental Table II. Medicare Benefits Schedule and Pharmaceutical Benefits Scheme items to be requested for data linkage

Supplemental Table III. Risk assessment and management by medical practitioners between the online course or text message intervention arms vs. control arm

Supplemental Table IV. Adverse events and serious adverse events for the online course or text message intervention arms vs. control arm

Supplemental Table V. SPIRIT 2013 Checklist

Supplemental Table VI. Statistical Analysis Plan (SAP) Checklist v 1.0 2019

**Supplemental Methods**

**Adverse event adjudication**

In our standardised operating procedure, we have defined SAE and AEs for this trial as follows:

A researcher blinded to group will provide an assessment of causality at the time of the initial report. Within the participant record in REDCap, one of the following categories should be assigned:

**Not related:** There is not a causal relationship between the study intervention and the adverse event. The event is due to an underlying or concurrent illness and is not related to the study intervention. There is another explanation for the event

**Probably not related:** The temporal association between the adverse event and study intervention is such that the study intervention is not likely to have any reasonable association with the adverse event.

**Possibly related:** The adverse event could have been caused by the study participant’s clinical state or the study intervention.

**Probably related:** The adverse event follows a reasonable temporal sequence from the time of study intervention or reappears when study intervention is reintroduced

**Supplemental Table I.** **Intervention Fidelity***

|  | **Operational element in the Love Your Brain study** |
| --- | --- |
| **Study Design** | - Standardised intervention with a clear protocol - Plan to include adherence to intervention in analysis (per-protocol analysis undertaken in addition to ITT analysis) |
| **Research team Training** | - Use of standardised training sessions - Quality assurance checks of process and content of training sessions - Role play of intervention follow-up telephone calls (for the third contact attempt only) - Further training delivered as required across study (e.g. change of staff) |
| **Delivery of Interventions** | - Use of a structured documentation process for delivery of interventions (email, text messages and online course) - Use of a form to capture the adaptations made in the delivery of the Love Your Brain intervention (allowing for identification, tracking and monitoring of adaptations to protocol) |
| **Receipt of Interventions** | - Receipt of all scheduled components (presented as the proportion of participants who received 100% of scheduled elements; as well as the mean level of completeness) - Audit of dispatch logs from the electronic messaging gateways (email, text messages and online course) |

******* *Consistent with Behaviour Change Consortium treatment fidelity recommendations (Bellg AJ, Borrelli, B et. Al. Health Psychol. 2004)*

**Supplemental Table II.** **Medicare** **Benefits Schedule and Pharmaceutical Benefits Scheme items to be requested for data linkage**

| **MBS item codes for determining primary outcome** | **Reason for request** |
| --- | --- |
| Chronic disease management items - (items 721 to 732) | Primary item of interest to indicate receipt of GP coordinated care |
| GP mental health treatment plan - (items 2700, 2701, 2715 or 2717) | Primary item of interest to indicate receipt of GP coordinated care |
| Heart Health Check (items 699 & 177) | Primary item of interest to indicate cardiovascular risk assessment |
| Attendances by general practitioners (excluding RACF attendances) - (items 3 to 19, 21 to 34, 36 to 42, 44 to 50, 58, 59, 60, 65, 193, 195, 197, 199, 597 to 600, 2497-2559, 5000-5067, 5220, 5223, 5227, 5228, 10992) | To assess frequency of GP attendance to determine regularity of contact |
| **MBS item codes for determining potential exclusion criteria** | **Reason for request** |
| Residential Aged Care Facility (RACF) Attendances - (items 20, 35, 43, 51, 92, 93 95, 96, 5010, 5028, 5049, 5067, 5260, 5263, 5265, 5267). | To identify registrants residing in an aged care facility |
| Medication management review in residential care – (item 903) | To identify registrants residing in an aged care facility |
| **MBS item codes for potential medical attendances** | **Reason for request** |
| Professional attendance by consultant physician – (items 116, 122,128, 131, 132, 133) | To account for regular medical attendances by a practitioner other than a GP |
| Other Medical practitioner attendances (non GP or GP to whom clause 2.3.1 applies) – (items 52, 53, 54, 57) | To account for regular medical attendances by a practitioner other than a GP |
| Other after hours attendances (non GP) – (items 5200 to 5208, 5220, 5223, 5227, 5228) | To account for regular medical attendances by a practitioner other than a GP |
| Specialist attendances other - (items 99, 104 to 109, 113, | To account for regular medical attendances by a practitioner other than a GP |
| Consultant physician attendances other - (items 110, 112, 114, 116, 117, 119, 120, 122, 128, 131, 132, 133) | To account for regular medical attendances by a practitioner other than a GP |
| Geriatrician referred patient assessment and management plan or attendance by a geriatrician - (items 141 to 147, 149) | To account for coordinated care type by a practitioner other than a GP |
| Attendances by Medical Practitioners who are Emergency Physicians - (Items 501 to 536) | To account for regular medical attendances by a practitioner other than a GP |
| Attendances by Medical Practitioners who are Public Health Physicians – (410 to 417) | To account for regular medical attendances by a practitioner other than a GP |
| Multidisciplinary case conferences by medical practitioners (other than specialist or consultant physician) - (items 735 to 758, 820 to 838, 855 to 872, 880)) | To account for coordinated care type by a practitioner other than a GP |
| Case conferences by consultant physician - (items 820 to 838, 6029 to 6034 and 6064 to 6075) | To account for coordinated care type by a practitioner other than a GP |
| Discharge case conference as part of hospital discharge – (items 830, 832, 834, 835, 837 and 838) | To determine if coordinated care has occurred within the hospital setting |
| Inpatient rehab case conferences - (item 880) | To identify those who were readmitted for inpatient rehab |
| Telehealth for medical practitioners providing clinical support for patients – (items 2100, 2122, 2125, 2126, 2137, 2138, 2143, 2147, 2179, 2195, 2199 and 2220) | Can account for medical attendances for patients living in rural areas. May be important for mode of delivery. |
| Telehealth specialist services – (items 99, 112, 149, 288, 389, 2820, 3015, 6016, 13210, 16399, 17609) and (items 113, 114, 384, 2799, 3003, 6004) | Can account for medical attendances for patients living in rural areas. May be important for mode of delivery. |
| Palliative medicine - (items 2801 to 3093) | To control for this as a confounder regarding hospital attendances and outcomes |
| Services provided by a practice nurse - (items 82200, 82205, 82210, 82215) | Can be used to provide health checks and coordinated care |
| Services provided a practice nurse registered as an Aboriginal Health Worker on behalf of the medical practitioner – (items 10983, 10984, 10987, 10997) | Can be used to provide health checks and coordinated care |
| Telehealth services provided by a practice nurse - (items 82220, 82221, 82222, 82223, 82224) | Can be used to provide health checks and coordinated care |
| Health Care Homes – (items 6087) | To account for coordinated care provided under an alternate model of care |
| **MBS item codes for treatments for prevention of stroke** | **Reason for request** |
| **Allied health items** |  |
| Audiologists – (item 10952) | Allied health service accessed through a Team care Arrangement |
| Chiropractors – (item 10964) | Allied health service accessed through a Team care Arrangement |
| Diabetes Educators – (item 10951) | Allied health service accessed through a Team care Arrangement |
| Dietitians - (item 10954) | Allied health service accessed through a Team care Arrangement |
| Exercise Physiologists – (item 10953) | Allied health service accessed through a Team care Arrangement |
| Mental Health Workers - (item 10956) | Allied health service accessed through a Team care Arrangement |
| Occupational Therapists - item 10958 | Allied health service accessed through a Team care Arrangement |
| Osteopaths – (item 10966) | Allied health service accessed through a Team care Arrangement |
| Physiotherapists – (item 10960) | Allied health service accessed through a Team care Arrangement |
| Podiatrists – (Item 10962) | Allied health service accessed through a Team care Arrangement |
| Psychologists – (item 10968) | Allied health service accessed through a Team care Arrangement |
| Speech Pathologists – (item 10970) | Allied health service accessed through a Team care Arrangement |
| Group Allied Health services for people with diabetes– (Items 81100 to 81125) | Allied health service accessed through a Team care Arrangement |
| Allied Health Services for people of Aboriginal or Torres Strait Islander descent – (Items 81100 to 81125) | Allied health service accessed |
| **Mental health items** |  |
| Consultant psychiatrist - initial consultations for new patients - (items 296 to 299 and 361) referred patient assessment and management plan (items 291, 293 and 359) and referral to allied mental health professionals | Coordinated care for those with mental health sequalae |
| Allied Mental Health Professionals psychological therapy services - (items 80000 to 80015, 80100 to 80115; 80125 to 80140; and 80150 to 80165) | Other psychological care for those with mental health sequalae |
| Mental Health group therapy services involving 6-10 patients (items 80020, 80120, 80145 and 80170) | Other psychological care for those with mental health sequalae |
| GP focussed psychological strategies (items 2721 to 2727) | Other psychological care for those with mental health sequalae |
| Telepsychiatry – (items 353 to 370) | Other psychological care for those with mental health sequalae |
| Consultant Psychiatry – (items 288, 291, 293, 296, 297, 299, 300, 302, 304, 306, 308, 310, 312, 314, 316, 318, 319, 320, 322, 324, 326, 328, 330, 332, 334, 336, 338, 342, 346, 348, 350, 352) | Other psychological care for those with mental health sequalae |
| Case Conferences by Consultant Psychiatrists - (items 855 to 866) | Form of psychological coordinated care |
| **Other medical treatment items** |  |
| Consultant occupational physician attendances (items 385 to 389) | Coordinated care for patients wishing to return to work. |
| Group therapy under supervision of medical practitioner - (items 170, 171, 172) | To assess frequency of GP attendance |
| Acupuncture by a GP – (items 173, 193, 195, 197, 199) |  |
| **MBS item codes for primary prevention management** | **Reason for request** |
| **Chemical investigations** |  |
| Items related to chemical investigations relevant to testing of cholesterol or glucose - (items 66500, 66503, 66506, 66509, 66512, 66536, 66539, 66542, 66551, 66557, 66560, 66841) | Chemical investigations relevant to prevention of stroke |
| **Health checks** |  |
| Health assessments (items 701, 703, 705, 707, 715) | Important for assessment of stroke risk factors for prevention |
| Health Assessment for Aboriginal and Torres Strait Islander People (MBS Item 715) | Important for assessment of stroke risk factors for prevention |
| Completion of the annual diabetes cycle of care for patients with established diabetes mellitus - (items 2517 - 2526 and 2620 - 2635) | Diabetes is a risk factor for stroke. This item is important for primary prevention |
| Medication management reviews - (items 900 [community]) | Important indicator for prevention of stroke |
| Miscellaneous diagnostic items – BP monitoring by an indwelling catheter (item 11600), ECG monitoring (11700, 11701, 11702, 11708, 11709, 11710, 11711, 11712) | Indicates care targeted at prevention of stroke |
| **PBS item codes** | **Reason for request** |
| ATC codes commencing with B01 (Antithrombotic agents) | To identify use of stroke prevention medications |
| ATC codes commencing with C (Cardiovascular system) | To identify use of stroke prevention medications |
| ATC codes commencing with N01 and N02 | To identify treatment of those with pain |
| ATC codes commencing with N05 and N06 | To identify treatment of those with mental health problems |

MBS: Medicare Benefits Schedule; PBS: Pharmaceutical Benefits Scheme; GP: General Practitioner; ATC: Anatomical Therapeutic Chemical.

**Supplemental Table III.** **Risk assessment and management by medical practitioners between the online course or text message intervention arms vs. control arm**

| **Assessment** | **Control (N=)** | | | **Online course (N=)** | | | **Text messages (N=)** | | | **Online vs**  **Control** | **Text vs Control** |
| --- | --- | --- | --- | --- | --- | --- | --- | --- | --- | --- | --- |
|  | **BL^a^**  **n (%)** | **W12^b^**  **n (%)** | **P value** | **BL^a^**  **n (%)** | **W12^b^**  **n (%)** | **P value** | **BL^a^**  **n (%)** | **W12^b^**  **n (%)** | **P value** | **RR***  **(95% CI)** | **RR***  **(95% CI)** |
| **Risk assessment undertaken at the visit** |  |  |  |  |  |  |  |  |  |  |  |
| Measure your blood pressure |  |  |  |  |  |  |  |  |  |  |  |
| Ask about your family history of stroke or heart attack |  |  |  |  |  |  |  |  |  |  |  |
| Send you for a blood test for cholesterol, kidney function, and/or blood sugar levels |  |  |  |  |  |  |  |  |  |  |  |
| 2 out of 3 above assessments undertaken |  |  |  |  |  |  |  |  |  |  |  |
| **Risk management undertaken at the visit** |  |  |  |  |  |  |  |  |  |  |  |
| Provide you with a care plan for managing your risk factors |  |  |  |  |  |  |  |  |  |  |  |

*BL: baseline; W12: 12 weeks ^a^:12 weeks immediately pre-randomisation; ^b^:12 weeks after randomisation; RR: Risk Ratios; CI: confidence interval; *adjusted for age and gender.*

**Supplemental Table IV.** **Procedure for definition and number of adverse events and serious adverse events for the online course or text message intervention arms vs. control arm**

|  | **Control**  **(N=)**  **n** | **Online Course**  **(N=)**  **n** | **Text Messages**  **(N=)**  **n** |
| --- | --- | --- | --- |
| Adverse events |  |  |  |
| Possibly Related to the intervention |  |  |  |
| Probably Related to the intervention |  |  |  |
| Serious Adverse events |  |  |  |
| Possibly Related to the intervention |  |  |  |
| Probably Related to the intervention |  |  |  |

**Supplemental Table V. SPIRIT 2013 Checklist**

| Section/item | ItemNo | Description | Page # |
| --- | --- | --- | --- |
| **Administrative information** | | |  |
| Title | 1 | Descriptive title identifying the study design, population, interventions, and, if applicable, trial acronym | 1 |
| Trial registration | 2a | Trial identifier and registry name. If not yet registered, name of intended registry | 2 |
|  | 2b | All items from the World Health Organization Trial Registration Data Set | N/A |
| Protocol version | 3 | Date and version identifier | 2 |
| Funding | 4 | Sources and types of financial, material, and other support | 10 |
| Roles and responsibilities | 5a | Names, affiliations, and roles of protocol contributors | 11 |
|  | 5b | Name and contact information for the trial sponsor | N/A |
|  | 5c | Role of study sponsor and funders, if any, in study design; collection, management, analysis, and interpretation of data; writing of the report; and the decision to submit the report for publication, including whether they will have ultimate authority over any of these activities | N/A |
|  | 5d | Composition, roles, and responsibilities of the coordinating centre, steering committee, endpoint adjudication committee, data management team, and other individuals or groups overseeing the trial, if applicable (see Item 21a for data monitoring committee) | 11 |
| Introduction |  |  |  |
| Background and rationale | 6a | Description of research question and justification for undertaking the trial, including summary of relevant studies (published and unpublished) examining benefits and harms for each intervention | 3-4 |
|  | 6b | Explanation for choice of comparators | 3 |
| Objectives | 7 | Specific objectives or hypotheses | 3 |
| Trial design | 8 | Description of trial design including type of trial (eg, parallel group, crossover, factorial, single group), allocation ratio, and framework (eg, superiority, equivalence, noninferiority, exploratory) | 3-4 |
| Methods: Participants, interventions, and outcomes | | |  |
| Study setting | 9 | Description of study settings (eg, community clinic, academic hospital) and list of countries where data will be collected. Reference to where list of study sites can be obtained | 6 |
| Eligibility criteria | 10 | Inclusion and exclusion criteria for participants. If applicable, eligibility criteria for study centres and individuals who will perform the interventions (eg, surgeons, psychotherapists) | 6-7 |
| Interventions | 11a | Interventions for each group with sufficient detail to allow replication, including how and when they will be administered | 3 |
|  | 11b | Criteria for discontinuing or modifying allocated interventions for a given trial participant (eg, drug dose change in response to harms, participant request, or improving/worsening disease) | 5-6 |
|  | 11c | Strategies to improve adherence to intervention protocols, and any procedures for monitoring adherence (eg, drug tablet return, laboratory tests) | 6 |
|  | 11d | Relevant concomitant care and interventions that are permitted or prohibited during the trial | N/A |
| Outcomes | 12 | Primary, secondary, and other outcomes, including the specific measurement variable (eg, systolic blood pressure), analysis metric (eg, change from baseline, final value, time to event), method of aggregation (eg, median, proportion), and time point for each outcome. Explanation of the clinical relevance of chosen efficacy and harm outcomes is strongly recommended | 7-8 |
| Participant timeline | 13 | Time schedule of enrolment, interventions (including any run-ins and washouts), assessments, and visits for participants. A schematic diagram is highly recommended (see Figure) | 9 |
| Sample size | 14 | Estimated number of participants needed to achieve study objectives and how it was determined, including clinical and statistical assumptions supporting any sample size calculations | 5 |
| Recruitment | 15 | Strategies for achieving adequate participant enrolment to reach target sample size | 6-7 |
| **Methods: Assignment of interventions (for controlled trials)** | | |  |
| Allocation: |  |  |  |
| Sequence generation | 16a | Method of generating the allocation sequence (eg, computer-generated random numbers), and list of any factors for stratification. To reduce predictability of a random sequence, details of any planned restriction (eg, blocking) should be provided in a separate document that is unavailable to those who enrol participants or assign interventions | 4 |
| Allocation concealment mechanism | 16b | Mechanism of implementing the allocation sequence (eg, central telephone; sequentially numbered, opaque, sealed envelopes), describing any steps to conceal the sequence until interventions are assigned | 4 |
| Implementation | 16c | Who will generate the allocation sequence, who will enrol participants, and who will assign participants to interventions | 4 |
| Blinding (masking) | 17a | Who will be blinded after assignment to interventions (eg, trial participants, care providers, outcome assessors, data analysts), and how | 4 |
|  | 17b | If blinded, circumstances under which unblinding is permissible, and procedure for revealing a participant’s allocated intervention during the trial | 4 |
| **Methods: Data collection, management, and analysis** | | |  |
| Data collection methods | 18a | Plans for assessment and collection of outcome, baseline, and other trial data, including any related processes to promote data quality (eg, duplicate measurements, training of assessors) and a description of study instruments (eg, questionnaires, laboratory tests) along with their reliability and validity, if known. Reference to where data collection forms can be found, if not in the protocol | 5-6 |
|  | 18b | Plans to promote participant retention and complete follow-up, including list of any outcome data to be collected for participants who discontinue or deviate from intervention protocols | 5-6 |
| Data management | 19 | Plans for data entry, coding, security, and storage, including any related processes to promote data quality (eg, double data entry; range checks for data values). Reference to where details of data management procedures can be found, if not in the protocol | 8-9 |
| Statistical methods | 20a | Statistical methods for analysing primary and secondary outcomes. Reference to where other details of the statistical analysis plan can be found, if not in the protocol | 7-9 |
|  | 20b | Methods for any additional analyses (eg, subgroup and adjusted analyses) | 8-9 |
|  | 20c | Definition of analysis population relating to protocol non-adherence (eg, as randomised analysis), and any statistical methods to handle missing data (eg, multiple imputation) | 6 |
| **Methods: Monitoring** | | |  |
| Data monitoring | 21a | Composition of data monitoring committee (DMC); summary of its role and reporting structure; statement of whether it is independent from the sponsor and competing interests; and reference to where further details about its charter can be found, if not in the protocol. Alternatively, an explanation of why a DMC is not needed | N/A |
|  | 21b | Description of any interim analyses and stopping guidelines, including who will have access to these interim results and make the final decision to terminate the trial | N/A |
| Harms | 22 | Plans for collecting, assessing, reporting, and managing solicited and spontaneously reported adverse events and other unintended effects of trial interventions or trial conduct | 9 |
| Auditing | 23 | Frequency and procedures for auditing trial conduct, if any, and whether the process will be independent from investigators and the sponsor | N/A |
| Ethics and dissemination | | |  |
| Research ethics approval | 24 | Plans for seeking research ethics committee/institutional review board (REC/IRB) approval | 9-10 |
| Protocol amendments | 25 | Plans for communicating important protocol modifications (eg, changes to eligibility criteria, outcomes, analyses) to relevant parties (eg, investigators, REC/IRBs, trial participants, trial registries, journals, regulators) | NA |
| Consent or assent | 26a | Who will obtain informed consent or assent from potential trial participants or authorised surrogates, and how (see Item 32) | 4 |
|  | 26b | Additional consent provisions for collection and use of participant data and biological specimens in ancillary studies, if applicable | N/A |
| Confidentiality | 27 | How personal information about potential and enrolled participants will be collected, shared, and maintained in order to protect confidentiality before, during, and after the trial | 10 |
| Declaration of interests | 28 | Financial and other competing interests for principal investigators for the overall trial and each study site | 10 |
| Access to data | 29 | Statement of who will have access to the final trial dataset, and disclosure of contractual agreements that limit such access for investigators | 10 |
| Ancillary and post-trial care | 30 | Provisions, if any, for ancillary and post-trial care, and for compensation to those who suffer harm from trial participation | N/A |
| Dissemination policy | 31a | Plans for investigators and sponsor to communicate trial results to participants, healthcare professionals, the public, and other relevant groups (eg, via publication, reporting in results databases, or other data sharing arrangements), including any publication restrictions | N/A |
|  | 31b | Authorship eligibility guidelines and any intended use of professional writers | N/A |
|  | 31c | Plans, if any, for granting public access to the full protocol, participant-level dataset, and statistical code | 10 |
| Appendices |  |  |  |
| Informed consent materials | 32 | Model consent form and other related documentation given to participants and authorised surrogates | N/A |
| Biological specimens | 33 | Plans for collection, laboratory evaluation, and storage of biological specimens for genetic or molecular analysis in the current trial and for future use in ancillary studies, if applicable | N/A |

*It is strongly recommended that this checklist be read in conjunction with the SPIRIT 2013 Explanation & Elaboration for important clarification on the items. Amendments to the protocol should be tracked and dated. The SPIRIT checklist is copyrighted by the SPIRIT Group under the Creative Commons “[Attribution-NonCommercial-NoDerivs 3.0 Unported](http://www.creativecommons.org/licenses/by-nc-nd/3.0/)” license

**Supplemental Table VI. Statistical Analysis Plan (SAP) Checklist v 1.0 2019**

| Section/Item | Index | Description | Reported on page # |
| --- | --- | --- | --- |
| **Section 1: Administrative information** | | | |
| Trial and Trial registration | 1a | Descriptive title that matches the protocol, with SAP either as a forerunner or subtitle,  and trial acronym (if applicable) | 1 |
|  | 1b | Trial registration number | 2 |
| SAP Version | 2 | SAP version number with dates | 2 |
| Protocol Version | 3 | Reference to version of protocol being used | 2 |
| SAP revisions | 4a | SAP revision history | 2 |
|  | 4b | Justification for each SAP revision | 2 |
|  | 4c | Timing of SAP revisions in relation to interim analyses, etc. | 2 |
| Roles and responsibility | 5 | Names, affiliations, and roles of SAP contributors | 11-12 |
| Signatures of: | 6a | Person writing the SAP | 11 |
|  | 6b | Senior statistician responsible | 11 |
|  | 6c | Chief investigator/clinical lead | 11 |
| **Section 2: Introduction** | | | |
| Background and rationale | 7 | Synopsis of trial background and rationale including a brief description of research question  and brief justification for undertaking the trial | 3 |
| Objectives | 8 | Description of specific objectives or hypotheses | 4 |
| **Section 3: Study Methods** | | | |
| Trial design | 9 | Brief description of trial design including type of trial (e.g., parallel group, multi-arm, crossover, factorial)  and allocation ratio and may include brief description of interventions | 4 |
| Randomization | 10 | Randomization details, e.g., whether any minimization or stratification occurred (including stratifying  factors used or the location of that information if it is not held within the SAP) | 4 |
| Sample size | 11 | Full sample size calculation or reference to sample size calculation in protocol  (instead of replication in SAP) | 5 |
| Framework | 12 | Superiority, equivalence, or noninferiority hypothesis testing framework, including which comparisons  will be presented on this basis | 5 |
| Statistical interim analysis and stopping guidance | 13a | Information on interim analyses specifying what interim analyses will be carried out  and listing of time points | NA |
|  | 13b | Any planned adjustment of the significance level due to interim analysis | NA |
|  | 13c | Details of guidelines for stopping the trial early | 5 |
| Timing of final analysis | 14 | Timing of final analysis, e.g., all outcomes analysed collectively or timing stratified  by planned length of follow-up | 5 |
| Timing of outcome assessments | 15 | Time points at which the outcomes are measured including visit “windows” | 6 |
| **Section 4: Statistical Principals** | | | |
| Confidence intervals and *P* values | 16 | Level of statistical significance | 6 |
|  | 17 | Description and rationale for any adjustment for multiplicity and, if so, detailing how the type 1 error  is to be controlled | NA |
|  | 18 | Confidence intervals to be reported | 6 |
| Adherence and Protocol deviations | 19a | Definition of adherence to the intervention and how this is assessed including extent  of exposure | 6 |
|  | 19b | Description of how adherence to the intervention will be presented | 6 |
|  | 19c | Definition of protocol deviations for the trial | 6 |
|  | 19d | Description of which protocol deviations will be summarized | 6 |
| Analysis populations | 20 | Definition of analysis populations, e.g., intention to treat, per protocol,  complete case, safety | 6 |
| **Section 5: Trial Population** | | | |
| Screening data | 21 | Reporting of screening data (if collected) to describe representativeness  of trial sample | 6 |
| Eligibility | 22 | Summary of eligibility criteria | 6 |
| Recruitment | 23 | Information to be included in the CONSORT flow diagram | 7 |
| Withdrawal/ Follow-up | 24a | Level of withdrawal, e.g., from intervention and/or from follow-up | 7 |
|  | 24b | Timing of withdrawal/lost to follow-up data | 7 |
|  | 24c | Reasons and details of how withdrawal/lost to follow-up data will be presented | 7 |
| Baseline patient characteristics | 25a | List of baseline characteristics to be summarized | 7 |
|  | 25b | Details of how baseline characteristics will be descriptively summarized | 7 |
|  | | | |
| Outcome definitions |  | List and describe each primary and secondary outcome including details of: | 7-8 |
|  | 26a | Specification of outcomes and timings. If applicable include the order of importance of primary  or key secondary end points (e.g., order in which they will be tested) | 8 |
|  | 26b | Specific measurement and units (e.g., glucose control, hbA1c [mmol/mol or %]) | 8 |
|  | 26c | Any calculation or transformation used to derive the outcome (e.g., change from baseline, QoL score,  Time to event, logarithm, etc.) | NA |
| Analysis methods | 27a | What analysis method will be used and how the treatment effects will be presented | 8 |
|  | 27b | Any adjustment for covariates | 8 |
|  | 27c | Methods used for assumptions to be checked for statistical methods | 8 |
|  | 27d | Details of alternative methods to be used if distributional assumptions do not hold, e.g., normality,  proportional hazards, etc. | 8 |
|  | 27e | Any planned sensitivity analyses for each outcome where applicable | 8 |
|  | 27f | Any planned subgroup analyses for each outcome including how subgroups are defined | 8 |
| Missing data | 28 | Reporting and assumptions/statistical methods to handle missing data (e.g., multiple imputation) | 8 |
| Additional analyses | 29 | Details of any additional statistical analyses required, e.g., complier-average causal effect10 analysis | 8 |
| Harms | 30 | Sufficient detail on summarizing safety data, e.g., information on severity, expectedness, and causality;  details of how adverse events are coded or categorized; how adverse event data will be analysed,  i.e., grade 3/4 only, incidence case analysis, intervention emergent analysis | 8-9 |
| Statistical software | 31 | Details of statistical packages to be used to carry out analyses | 9 |
| References | 32a | References to be provided for nonstandard statistical methods | 10 |
|  | 32b | Reference to Data Management Plan | 10 |
|  | 32c | Reference to the Trial Master File and Statistical Master File | NA |
|  | 32d | Reference to other standard operating procedures or documents to be adhered to | 10 |

**Taken from the paper:** Gamble C, Krishan A, Stocken D, Lewis S, Juszczak E, Doré C, et al. Guidelines for the Content of Statistical Analysis Plans in Clinical Trials. JAMA. 2017;318(23):2337-43.

**Abbreviations:** CONSORT, Consolidated Standards of Reporting Trials; hbA1c, haemoglobin A1c; QoL, quality of life; SAP, statistical analysis plan.

For more information visit:

*The development of this checklist was funded by the* [*MRC Hubs for Trials Methodology Research*](https://www.methodologyhubs.mrc.ac.uk/)
